# Molecular Insight into the Role of HLA Genotypes in Immunogenicity and Secondary Refractoriness to Anti-TNF Therapy in IBD Patients

**DOI:** 10.3390/ijms26157274

**Published:** 2025-07-28

**Authors:** Mladen Maksic, Irfan Corovic, Tijana Maksic, Jelena Zivic, Milos Zivic, Natasa Zdravkovic, Aleksa Begovic, Marija Medovic, Djordje Kralj, Zeljko Todorovic, Milica Cekerevac, Rasa Medovic, Milos Nikolic

**Affiliations:** 1 Department of Internal Medicine, Faculty of Medical Sciences, University of Kragujevac, Svetozara Markovica 69, 34000 Kragujevac, Serbia; asussonicmaster95@gmail.com (M.M.); jelena.zy@gmail.com (J.Z.); natasasilvester@gmail.com (N.Z.); todorovic_zeljko@hotmail.com (Z.T.); 2 Clinic of Gastroenterology and Hepatology, University Clinical Centre Kragujevac, Zmaj Jovina 30, 34000 Kragujevac, Serbia; 3 Center for Molecular Medicine and Stem Cell Research, Faculty of Medical Sciences, University of Kragujevac, Svetozara Markovica 69, 34000 Kragujevac, Serbia; ira.corovic@gmail.com; 4 Department of Internal Medicine, General Hospital of Novi Pazar, Generala Zivkovica 1, 36300 Novi Pazar, Serbia; 5 Department of Pediatrics, Faculty of Medical Sciences, University of Kragujevac, Svetozara Markovica 69, 34000 Kragujevac, Serbia; tijanaveljkovic96@gmail.com (T.M.); milicacekerevac@gmail.com (M.C.); rasamedovic@gmail.com (R.M.); 6 Pediatric Clinic, University Clinical Centre Kragujevac, Zmaj Jovina 30, 34000 Kragujevac, Serbia; 7 Department of Dentistry, Faculty of Medical Sciences, University of Kragujevac, Svetozara Markovica 69, 34000 Kragujevac, Serbia; zivicmilos5@gmail.com; 8 Department of Maxillofacial Surgery, Clinic of Otorhinolaryngology, University Clinical Centre Kragujevac, Zmaj Jovina 30, 34000 Kragujevac, Serbia; 9 Digestive Endoscopy Unit, University Clinic Dr Dragisa Misovic-Dedinje, 11000 Belgrade, Serbia; alexa13begovic@gmail.com; 10 Department of Dermatovenerology, Faculty of Medical Sciences, University of Kragujevac, Svetozara Markovica 69, 34000 Kragujevac, Serbia; makastojanovic88@gmail.com; 11 Centre for Dermatovenerology, University Clinical Centre Kragujevac, Zmaj Jovina 30, 34000 Kragujevac, Serbia; 12 Department of Gastroenterology, University Hospital Medical Center Zvezdara, 11000 Belgrade, Serbia; drkraljdjordje@gmail.com; 13 Clinic of Hematology, University Clinical Centre Kragujevac, Zmaj Jovina 30, 34000 Kragujevac, Serbia; 14 Department of Pharmacy, Faculty of Medical Sciences, University of Kragujevac, Svetozara Markovica 69, 34000 Kragujevac, Serbia

**Keywords:** HLA genotypes, inflammatory bowel disease, ulcerative colitis, Crohn’s disease, anti-TNF therapy, immunogenicity, pharmacogenetics, anti-drug antibodies, HLA-DQA1*05, precision medicine

## Abstract

The emergence of anti-TNF agents has revolutionized the management of inflammatory bowel disease, yet a significant proportion of patients experience primary non-response or secondary loss of response due to immunogenicity. As the field of precision medicine advances, genetic predictors such as human leukocyte antigen (HLA) variants are gaining increasing attention. This review provides a comprehensive synthesis of current evidence on the role of HLA genotypes in inflammatory bowel disease susceptibility and disease behavior, with a focus on their mechanistic and clinical relevance in anti-TNF therapy. Special emphasis is placed on HLA-DQA1*05, a validated predictor of anti-drug antibody formation and reduced therapeutic durability. We explore the immunological basis of HLA-mediated immunogenicity, summarize pharmacogenetic and biomarker findings, and discuss how HLA typing may be integrated into treatment algorithms to improve patient stratification and long-term outcomes. As immunogenetics continues to inform clinical decision-making, understanding the interplay between HLA polymorphisms and therapeutic response offers new opportunities for biomarker-guided, personalized care in inflammatory bowel disease.

## 1. Introduction

Inflammatory bowel disease (IBD), encompassing Crohn’s disease (CD) and ulcerative colitis (UC), is a multifactorial disorder arising from complex interactions between environmental triggers, intestinal microbiota, and dysregulated immune responses in genetically susceptible individuals. Twin and family studies have unequivocally demonstrated a genetic component in the pathogenesis of IBD, evidenced by the significant risk of developing IBD if a family member is affected [1]. Advancements in genotyping and genome-wide association studies (GWASs) have demonstrated that IBD is fundamentally a polygenic disease, identifying 242 risk loci [2]. Moreover, advancements in computational methods, such as machine learning and deep learning, have significantly contributed to the identification of new disease mechanisms. Using the Support Vector Machine method, 231 new genes associated with IBD were identified [3]. Among the strongest genetic contributors are variants within the HLA region [4]. In UC, the HLA-DRB1*01:03 allele shows consistent associations with disease severity [5,6], whereas CD demonstrates more heterogeneous HLA signals and a stronger influence from non-HLA genes such as NOD2 and ATG16L1 [7]. Notably, the role of HLA genotypes is expanding beyond disease susceptibility toward shaping therapeutic outcomes. Anti-TNF agents, including infliximab and adalimumab, remain a cornerstone of IBD treatment. However, primary non-response and secondary loss of response (LOR) affect up to 50% of patients, often due to the formation of anti-drug antibodies (ADAs) [8]. Recent evidence implicates the HLA-DQA1*05 allele in increased immunogenicity to anti-TNF therapies, positioning it as a promising biomarker for treatment stratification [9,10].

Immunological defense mechanisms, self-tolerance, and the development of immunologically mediated illnesses are all dependent on the HLA system, which serves as the primary mechanism for immune control and antigen recognition. Additionally, the major histocompatibility complex (MHC), which is located on chromosome 6p21, is responsible for its encoding [11]. HLA research has evolved to include a more thorough understanding of its role in autoimmune disorders, infectious diseases, and chronic inflammatory disorders. This expansion from its function in organ transplantation has been a driving force behind this expansion. There are a number of well-known disease categories in which HLA has shown significant relevance. One of these categories is IBD, which includes CD and UC [12,13].

Class I (HLA-A, -B, and -C), class II (HLA-DR, -DQ, and -DP), and class III are the three classes that are generally used to categorize the HLA complex [11]. Endogenous peptides, which are predominantly produced from intracellular pathogens, are presented to CD8+ cytotoxic T lymphocytes by HLA class I molecules, which are expressed on nearly all nucleated cells. On the other hand, HLA class II molecules are mostly expressed on professional antigen-presenting cells, including dendritic cells, macrophages, and B cells. These cells are responsible for presenting foreign peptides to CD4+ helper T cells [14]. In spite of the fact that it is not involved in antigen presentation, the class III region is responsible for encoding molecules that are crucial for immunological signaling. These molecules include components of the complement cascade as well as pro-inflammatory cytokines like TNF-α [15]. Both class I and class II HLA molecules are heterodimeric glycoproteins as far as their structural makeup is concerned. Class I molecules are made up of a heavy α-chain that is non-covalently coupled with β2-microglobulin. This heavy α-chain possesses three extracellular domains, namely α1, α2, and α3. It is the α1 and α2 domains that come together to form the peptide-binding groove, which is capable of accommodating peptides that are between 8 and 10 amino acids in length [14,15]. Two transmembrane chains, namely α and β, are the constituents of class II molecules. Each of these chains possesses two extracellular domains. It is the α1 and β1 domains that are responsible for forming the peptide-binding groove. This groove is open at both ends, which enables it to accommodate longer peptides, which typically consist of 13–25 amino acids [16]. T-cell recognition and antigen processing are both determined by this structural variation, which also determines how antigens are presented and processed [11,14]. Moreover, the glycosylation of HLA molecules is a factor that contributes to the stability of these molecules, as well as their shape and interactions with other immune receptors. Modifications to glycoproteins have the potential to influence immunogenicity as well as the dynamics of surface expression that occur under healthy and pathological settings [12]. HLA genes are characterized by a significant amount of polymorphisms, notably in the sections of class I and II molecules that are responsible for binding peptide bonds. This genetic heterogeneity makes it easier to provide a wide variety of antigenic peptides and contributes to the efficient surveillance of the immune system during immunological response. More than 30,000 different HLA alleles have been discovered up to this point, with HLA-B being the gene in the human genome that possesses the highest degree of polymorphisms [17,18]. These allelic changes have the potential to change the affinity and specificity of peptide binding, which can result in individual variances in susceptibility or resistance to infections, autoimmune disorders, and responses to treatment. It is also important to note that this polymorphism is subject to significant selective pressure from pathogens, which is a factor that contributes to its evolutionary persistence and functional significance across populations [18,19].

This review aims to synthesize current knowledge on the molecular, immunogenetic, and clinical implications of HLA variants, particularly HLA-DQA1*05, in IBD patients receiving anti-TNF therapy, with a focus on refractoriness, immunogenicity, and prospects for precision medicine.

## 2. HLA in Autoimmune Diseases

**Figure 1:**
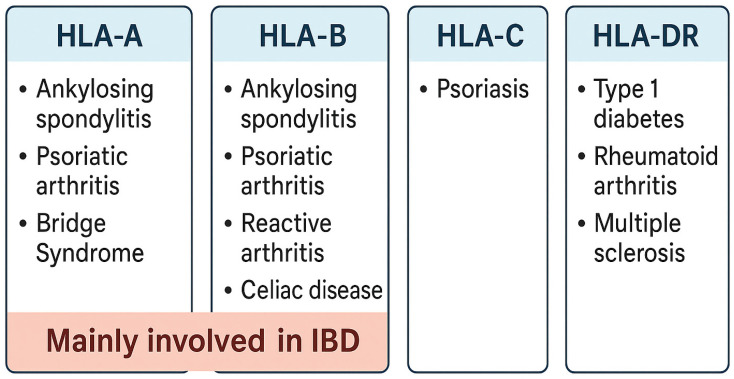
Types of HLAs and their associated diseases. This diagram categorizes different HLA gene classes (HLA-A, HLA-B, HLA-C, and HLA-DR) and their associated autoimmune or inflammatory diseases. It highlights the major associations, such as HLA-B with ankylosing spondylitis and celiac disease, HLA-C with psoriasis, and HLA-DR with type 1 diabetes and multiple sclerosis. A special note is made that certain HLA types, especially HLA-B, are mainly involved in inflammatory bowel disease (IBD).

The link between specific HLA alleles and autoimmune diseases is well established. In the context of immune-mediated diseases, this connection is widely used as a model for gene–environment interactions [13]. Certain examples include the HLA-DR3 and DR4 alleles, which have been associated with type 1 diabetes mellitus [20]; the HLA-B27 allele, which has been linked to ankylosing spondylitis [21]; and the HLA-DQ2/DQ8 variant, which has been linked to celiac disease (Figure 1) [13]. There are a number of mechanisms that are considered to be responsible for the establishment of these links. Some of these mechanisms include the presentation of cryptic self-antigens, the failure of negative selection in the thymus, and the phenomenon known as molecular mimicry, in which foreign antigens resemble self-peptides. Furthermore, certain alleles may deliver peptides in a conformation that modifies the repertoire of T-cell receptors (TCRs) or co-stimulatory signals, thereby shifting the equilibrium in favor of autoimmunity [13,22]. The genetic links in question are not merely statistical in nature; rather, they typically correspond with particular clinical presentations, the progression of the disease, and the responses to treatment. The presence of HLA-B27 in spondyloarthropathies, for instance, is linked to an earlier beginning of the disease, involvement of the axial region, and a responsiveness to TNF inhibitors during the course of the medical condition [21,23,24,25]. Therefore, it is vital to have a solid understanding of the mechanisms that are mediated by HLA in order to acquire insights into immunopathology as well as disease prediction.

## 3. HLA Associations in Inflammatory Bowel Disease

IBD is a chronic inflammatory disorder of the gastrointestinal tract with a multifactorial etiology. The development and progression of this disease are affected by various factors, such as environmental exposure, gut microbiota, and immune system dysregulation; nonetheless, genetic predisposition remains a critical element [1]. Over two hundred risk loci associated with IBD have been identified by GWASs, many of which pertain to immune regulatory pathways [2]. The HLA region is notable for its consistent connection, especially in UC.

### 3.1. HLA in Ulcerative Colitis

UC is strongly associated with specific HLA class II alleles, particularly those within the HLA-DRB1 locus [26]. Among individuals with UC, the HLA-DRB1*01:03 allele, previously linked to disease incidence, has also been associated with more severe clinical outcomes, including an increased risk of hospitalization, use of systemic corticosteroids, and the need for major surgical intervention [27,28]. Moreover, an increased frequency of DRB113 alleles (including *1309, *1320, *1325, and *1329) has been observed in an Italian cohort of patients with pancolitis, those undergoing surgical resection, and individuals presenting with extraintestinal manifestations [29].

In Japanese patients with UC, HLA-DRB1*08 has been significantly associated with more extensive disease beyond the rectum, while HLA-DRB1*09 has been linked to a later age at diagnosis, suggesting that HLA-DRB1 influences not only disease susceptibility but also clinical phenotype [30]. HLA-DRB1*1502 is consistently associated with UC across European, North American, Japanese, and Korean populations, with a similar relative risk despite wide variation in allele frequency, showing the highest in Japanese and lowest in Northern European populations [31]. In pediatric-onset UC, the HLA region accounts for nearly the entire genetic association, with HLA-DRB1*01:03 showing the strongest link, significantly exceeding its effect in adult-onset UC; additional independent associations have been identified with HLA-DRB1*13:01 and SNP rs17188113, suggesting both shared and pediatric-specific genetic drivers, particularly related to extensive colitis and female gender, highlighting the central role of antigenic stimulation in early disease development [31]. Regarding extraintestinal manifestations, HLA-DRB1*01:03 shows the strongest association with type I arthritis and uveitis, while HLA-B44 and TNF-1031C are linked to type II arthritis and erythema nodosum, though these associations may partly reflect colonic disease involvement [26].

Notably, HLA associations in UC are more robust and consistent across different ethnic populations compared to CD, reinforcing the hypothesis that UC pathogenesis is more tightly linked to antigen presentation pathways.

### 3.2. HLA in Crohn’s Disease

Multiple studies have demonstrated significant, though often population-specific, associations between HLA class II alleles and CD. In the Japanese population, the allele HLA-DRB1*0450 is positively associated with CD, while DRB1*1502 shows a protective effect [32]. Similar patterns exist in other ethnic groups, though results remain partially inconsistent due to methodological and demographic variability. Certain haplotypes (e.g., DRB1*0405-DQB1*0401) and non-classical MHC genes such as HLA-G and MICA/MICB have also been linked to specific CD phenotypes, including fistulizing disease and ileocecal involvement [33]. Additionally, HLA-DRB1*0103 and HLA-B*44 are associated with extraintestinal manifestations like arthritis and uveitis [34,35]. Finally, emerging evidence suggests that HLA and other immune-related polymorphisms may influence disease course, phenotype, and response to biologic therapies, highlighting their potential role in personalized treatment approaches for CD [33]. The co-occurrence of HLA-B27-associated spondyloarthropathies in CD suggests shared pathogenic pathways driven by class I-mediated cytotoxic responses and aberrant innate immunity [36,37]. Importantly, unlike UC, genetic risk in CD is more strongly influenced by non-HLA loci, including NOD2, IL23R, and ATG16L1, which play pivotal roles in microbial sensing, autophagy, and epithelial barrier function [38]. This highlights the broader genetic architecture of CD, where both innate and adaptive immune pathways contribute to disease pathogenesis.

Table 1 provides a comprehensive summary of HLA alleles and haplotypes associated with IBD, highlighting their relevance to disease susceptibility, clinical phenotype, and extraintestinal manifestations in both UC and Crohn’s disease.

### 3.3. Role of HLA in Shaping IBD Precision Medicine

**Figure 2:**
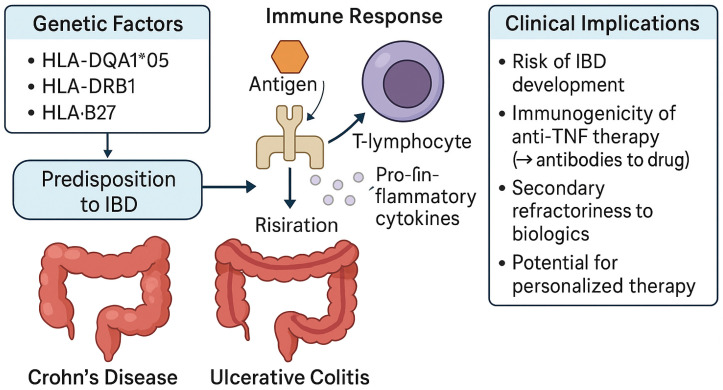
The role of HLA genes in IBD pathogenesis and second loss response. This schematic summarizes the contribution of HLA gene variants (HLA-DQA1*05, HLA-DRB1, and HLA-B27) to the pathogenesis of inflammatory bowel disease (IBD), including Crohn’s disease and ulcerative colitis. It illustrates how genetic predisposition influences immune responses, particularly T-lymphocyte activation and the release of pro-inflammatory cytokines, leading to chronic inflammation. The right panel outlines clinical implications such as increased risk of IBD development, immunogenicity to anti-TNF therapy (via antibody formation), secondary refractoriness to biologics, and the potential for personalized treatment strategies.

While HLA typing is not yet a part of routine clinical assessment in IBD, its growing relevance is becoming apparent, especially in the context of treatment response and resistance. One of the most critical emerging themes in IBD management is the identification of genetic predictors of therapeutic refractoriness and the development of resistance to biologic therapies. Certain HLA class II alleles, such as the HLA-DQA1*05 allele, had a significantly higher risk of developing ADAs and treatment failure when receiving infliximab or adalimumab (Figure 2) [39]. Evidence is accumulating that specific HLA haplotypes may influence the pharmacodynamics of biologics and small molecules, potentially modifying efficacy, durability of response, and risk of adverse effects [40,41]. This refractoriness not only complicates clinical management but also contributes to disease progression, increased healthcare utilization, and reduced quality of life. Thus, HLA genotyping may emerge as a valuable tool in the personalization of IBD therapy, enabling the prediction of biologic responsiveness, guiding early therapeutic decisions, and improving long-term disease control. Importantly, the potential of HLA to serve as a biomarker of treatment failure offers an opportunity to shift from reactive to proactive disease management [42,43].

## 4. Genetic Modulation of Anti-TNF Pharmacokinetics in IBD

The anti-TNF drugs currently approved by the FDA include four monoclonal antibodies and one fusion protein that functions as a TNFα receptor [44,45]. Among anti-TNF drugs, infliximab and adalimumab are the most commonly used biologics for IBD therapy [46,47]. Although these drugs have demonstrated significant clinical efficacy, therapeutic responses among IBD patients remain highly variable. Namely, approximately 30% of patients exhibit a primary non-response, failing to achieve initial clinical benefit, while up to 50% of patients discontinue treatment over time due to secondary LOR or the development of adverse effects [48,49]. The pharmacokinetics of biologic agents have garnered significant attention in recent years, given their critical influence on long-term therapeutic outcomes. A comprehensive knowledge of factors influencing early drug concentrations can lead to early optimization and improved clinical efficacy of biologics. The absorption of anti-TNF agents largely depends on the route of administration, whereas their elimination is minimally influenced by renal and biliary pathways, as is the case with other IgG antibodies [50]. However, the metabolic mechanisms underlying the clearance of anti-TNF drugs remain poorly understood [51,52]. Previous studies have shown that more than 90% of patients carry at least one pharmacogenetic variant that may affect both the pharmacokinetics and pharmacodynamics of a specific biological drug [53]. Due to higher specificity for biological targets, TNF inhibitors differ from small-molecule inhibitors in their pharmacokinetic profiles [54]. Importantly, the relationship between dosing regimens and serum concentrations of biologics is nonlinear and is influenced by a variety of factors. Several factors are known to influence the pharmacokinetics of anti-TNF agents, with the development of ADAs having the most significant impact. In addition, various patient- and disease-related characteristics such as gender, body size, concomitant use of immunomodulators, type of underlying disease, serum albumin levels, and the degree of systemic inflammation have also been shown to affect serum drug levels [50]. Several pharmacokinetic-modifying factors have been associated with specific forms of IBD. For example, patients with ulcerative colitis have been shown to exhibit lower infliximab trough concentrations compared to those with Crohn’s disease. This difference is likely due to the more extensive mucosal inflammation in ulcerative colitis, which leads to increased intestinal permeability and increased fecal loss of infliximab [55]. Body mass index is another factor known to influence the clearance of monoclonal antibodies in a nonlinear manner. Although weight-based dosing regimens may risk underdosing patients with low body weight, evidence suggests that infliximab clearance also increases with higher body weight. This may be related to the pro-inflammatory role of mesenteric fat and its associated increase in TNFα production [56].

A relationship between pharmacogenomic variants and the clinical response to biologics has been well established. Single-nucleotide polymorphisms (SNPs) influencing infliximab trough concentrations have been shown to contribute to substantial interindividual variability in drug bioavailability. Specifically, lower trough levels of infliximab have been observed in patients carrying the *ATG16L1* gene variants rs7587051 and rs143063741 (GG genotypes), the *C1orf106* variant rs442905 (GA genotype) [57], and the rs763110 allele in the *FASLG* gene [58]. A study by Juanola et al. investigated three SNPs in the *NOD2* gene (rs2066844 (T), rs2066845 (C), and rs2066847 (C)) and found that carriers of any of these variants were more likely to require intensified TNF inhibitor regimens due to secondary LOR [59]. Moreover, Schäffler et al. analyzed the combined effect of the three previously mentioned SNPs and indicated that the presence of the variant allele is associated with lower trough concentrations [60]. On the other hand, the *C1orf106* variant rs59457695 (CC genotype) [57] and the IL6 variant rs10499563 (CC genotype) [61] have demonstrated a significant association with higher trough concentrations of infliximab. In a study by Salvadó-Martín et al., SNP rs1816702 in the *TLR2* gene was identified as a potential biomarker for guiding treatment decisions in pediatric IBD patients. This SNP, along with four others, *TLR4g*.117712004 (rs5030728), *LY96g*.73989727 (rs11465996), *TNFRSF1Bg*.12207235 (rs3397), and *CD14g*.140633331 (rs2569190), was associated with below-therapeutic, supertherapeutic, or non-detectable trough concentrations of adalimumab or infliximab. Moreover, patients carrying these alleles demonstrated differential pharmacokinetic responses to the same biologic drug. In that sense, carriers of the T allele at *TLR2* rs1816702 or the G allele at *CD14* rs2569190 exhibited higher serum concentrations of adalimumab, while individuals with the C allele at *TNFRSF1B* rs3397 had lower serum levels of this drug. The same research results also suggest that patients with the TT genotype at *TLR2* rs1816702 may benefit more from adalimumab than infliximab [62].

An Italian cohort study demonstrated a significant association between the *FCGR3A* gene variant and median infliximab concentrations during maintenance therapy. Specifically, patients with the wild-type genotype exhibited higher infliximab levels compared to those carrying the variant allele, indicating a potential pharmacogenetic influence on drug exposure [63]. Inadequate serum infliximab concentrations resulting from pharmacokinetic failure represent the primary cause of treatment failure in pediatric patients with IBD. Standard dosing regimens of anti-TNFα agents often fail to achieve therapeutic trough concentrations in children, particularly in the presence of hypoalbuminemia [64]. Kelsen et al. reported that children with Crohn’s disease exhibited lower response rates to infliximab compared to older pediatric patients [65]. More recent evidence indicates that younger children require significantly higher weight-based doses of infliximab to maintain clinical remission and achieve therapeutic trough levels. Furthermore, this younger age group is at increased risk of developing ADA, likely due to subtherapeutic trough concentrations during the induction phase and early maintenance therapy [66]. Low body weight is another factor contributing to inadequate infliximab exposure. Drug clearance is not linearly related to weight, making low-weight patients, particularly those with infantile-onset IBD, especially vulnerable to underdosing. Body surface area has also been identified as an important determinant of anti-TNFα pharmacokinetics, with younger children having a higher BSA-to-weight ratio that may influence drug distribution and clearance [67].

Genetic variability in both the Fc [68] and Fc gamma receptors [26] can significantly influence the pharmacokinetics of TNF inhibitors. Notably, two of the five alleles within a variable number tandem repeat (VNTR) region of the *FCGRT* gene have been associated with lower serum levels of TNF inhibitors at the onset of treatment. Patients with the VNTR2/VNTR3 genotype had reduced infliximab or adalimumab concentrations compared to those with the VNTR3/VNTR3 genotype [69]. Furthermore, polymorphisms in Fc gamma receptors may contribute to enhanced clearance of monoclonal antibodies by increasing the binding affinity of their Fc regions to high-affinity Fc gamma receptors. This interaction facilitates more rapid elimination, leading to decreased serum drug levels and potentially diminished therapeutic efficacy [70].

Although certain genetic polymorphisms may initially appear promising, larger cohorts and additional statistical corrections are often necessary to validate them and confirm their true clinical significance in pharmacokinetic–pharmacodynamic modeling of biologics. Pharmacokinetic modeling can be employed to design optimal dosing regimens for biologics and to support individualized decision-making, especially in vulnerable patients.

A summary of pharmacogenetic polymorphisms influencing anti-TNF pharmacokinetics is provided in Table 2.

## 5. Contribution of HLA-DQ Alleles to Immunogenicity and Resistance to Anti-TNF Therapy

Over the past three decades, the pharmaceutical industry has undergone a significant shift toward the development and use of biological therapies. Among these, monoclonal antibodies (mAbs) emerged as the most extensively used class [71]. Despite the availability of newer therapeutic options, anti-TNF-α agents remain the cornerstone of initial biologic treatment in inflammatory bowel disease (IBD). Infliximab (IFX) and adalimumab (ADA) are approved for both Crohn’s disease (CD) and ulcerative colitis (UC), with IFX showing superior efficacy when combined with immunosuppressants [72,73]. However, treatment failure may occur due to the development of ADAs, which compromise efficacy and increase adverse events. While the immunological basis of ADA formation is not fully understood, early identification of high-risk patients could offer clinical advantages. Loss of therapeutic response—either primary or secondary—is often linked to immunogenicity, where a reciprocal relationship exists between subtherapeutic drug levels and ADA production, likely due to enhanced drug clearance. The risk of immunogenicity can be reduced through concomitant use of immunomodulators such as methotrexate or thiopurines [74]. The HLA complex plays a crucial role in immune tolerance regulation and the adaptive immune response. One of the complex mechanisms implicated in the etiopathogenesis of IBD involves immune dysregulation in response to commensal or pathogenic bacteria, potentially modulated by specific HLA gene variants. The HLA complex, the human equivalent of the major histocompatibility complex (MHC), is a highly polymorphic gene family. The HLA-DQA1 gene belongs to the MHC class II subgroup, which is primarily involved in the presentation of extracellular antigens to CD4+ T lymphocytes. Hundreds of HLA-DQA1 alleles have been identified, each denoted by a specific nomenclature, with HLA-DQA105:01 being common, particularly in 40% of European individuals [75]. Interactions between HLA-DQA1 variants and other HLA genes have been linked to susceptibility to various autoimmune diseases, including rosacea, narcolepsy, idiopathic inflammatory myopathies, alopecia areata, juvenile idiopathic arthritis, and autoimmune Addison’s disease. Specifically, carrying the HLA-DQA1*05 allele has been associated with an increased risk for disorders such as celiac disease and type 1 diabetes, while also being linked to a reduced risk of developing rheumatoid arthritis [76,77,78]. These associations underscore the potential role of HLA-DQA1 allelic variation in shaping dysregulated adaptive immune responses.

The precise mechanisms by which different allelic variants of the HLA-DQA1 gene contribute to autoimmune disease risk are not fully understood. A major challenge in elucidating these mechanisms is the strong linkage disequilibrium within the HLA region, leading to the co-inheritance of specific alleles that can confound genetic association studies. HLA-DQA105:05 is commonly inherited with HLA-DQB103:01 and HLA-DRB1*11:01. Co-inheritance patterns within the HLA region are important for the molecular basis of HLA-associated immunologically mediated adverse drug reactions. HLA class II molecules, heterodimers composed of an alpha and a beta chain, are analyzed based on genetic variation in only one constituent gene. This limits the ability to determine which specific alpha–beta chain combinations confer the greatest immunological risk. Autoimmune disorders result from a complex interplay of genetic predispositions and environmental triggers. Among the genetic factors, the HLA-DQA105 allele has been identified as a potential contributor to immunogenicity in patients receiving anti-TNF therapies such as infliximab IFX and ADA. Emerging evidence indicates a strong association between carrying the HLA-DQA105 allele and the development of ADAs in some patients with IBD, irrespective of whether biologic therapy is used as monotherapy or in combination with immunomodulators [79]. The HLA-DQA105 allele has been associated with increased immunogenicity and an elevated risk of loss of therapeutic response to anti-TNF agents in CD and UC. The strength of this association and its clinical relevance may differ between these diseases, suggesting potential subtype-specific implications for personalized treatment strategies. The HLA-DQ molecule presents TNF-blocking peptides to T cells, initiating an immune response that leads to ADA production and neutralization of anti-TNF therapy [39]. Some studies suggest a stronger association in CD, especially among patients treated with IFX.

Wang et al. conducted a genotypic analysis to assess the association between HLA-DQA105 variants and clinical outcomes in CD patients treated with IFX. Among 345 patients, 17 experienced IFX-related adverse events, including infusion reactions, infections, psoriasiform rash, anaphylactoid responses, and certain tumors. While no significant difference in overall adverse event rates was observed between HLA-DQA105G variant carriers and wild-type individuals, ADA development was significantly more frequent in variant carriers, indicating a genetic predisposition to immunogenicity. This association remained significant after adjusting for confounders such as age, sex, body weight, and immunomodulator use. Furthermore, HLA-DQA105 wild-type carriers receiving IFX combined with immunomodulators achieved higher clinical remission rates compared to those on IFX monotherapy or variant carriers [80]. These findings suggest that the HLA-DQA105 genotype may influence both immunogenicity and therapeutic response to IFX in CD.

In an observational genome-wide association study involving biologically naïve patients with CD, Sazonovs et al. identified a significant association between the HLA-DQA105 allele and the development of ADAs against anti-TNF agents. Their analysis revealed the highest immunogenicity rate—92% at one year—among patients treated with infliximab monotherapy who carried the HLA-DQA105 allele. In contrast, the lowest immunogenicity rate, 10% at one year, was observed in patients receiving adalimumab combination therapy who did not carry the allele [39]. Complementary findings from a recent Oxford study further refined this genetic risk: individuals with IBD who carried HLA-DQA1 05:05 were found to be at an increased risk of developing antibodies against both infliximab and adalimumab, whereas the HLA-DQA1 05:01 allele was associated only with antibody formation against infliximab and not adalimumab [81].

Reppell et al. reported that the strongest genetic association with antibody development in response to ADA therapy was observed with HLA-DQA1 05:05 and DRB1 01:02 in both CD and UC patients. Their study reaffirmed that HLA-DQA1 05:01 was not associated with the development of antibodies to ADA [82]. Two recent studies have investigated the impact of the HLA-DQA105 allele in patients with IBD undergoing anti-TNF therapy guided by proactive therapeutic drug monitoring (pTDM). Both studies reported a low incidence of immunogenicity, which was primarily attributed to subtherapeutic drug levels and failure to achieve target drug concentrations, rather than to HLA-DQA1 05 status [83,84]. These findings suggest that pTDM may mitigate the immunogenic risk associated with HLA-DQA105, likely through maintenance of therapeutic drug levels. Some data indicate that the development of ADAs was associated with more frequent subtherapeutic anti-TNF concentrations and that these subtherapeutic levels occurred more frequently in HLA-DQA105 carriers [83]. Additional studies have reported that immunogenicity in HLA-DQA1*05 carriers may be attenuated by the concurrent use of immunosuppressive agents, such as thiopurines or methotrexate [39,84]. Unlike anti-TNF agents, the HLA-DQA105 allele does not affect response to biologics such as vedolizumab or ustekinumab [85]. The role of this allele in patients treated with Janus kinase (JAK) inhibitors and anti-interleukin-23 (IL-23) antagonists remains unclear. Other genetic variants, including FCGR3A11 and HLA-DRB112/*13, have been proposed as potential contributors to increased immunogenicity in response to anti-TNF, though they have not achieved genome-wide significance.

Overall, these findings highlight the potential clinical benefit of integrating genetic and pharmacological data to personalize treatment strategies in IBD. For patients at high genetic risk of immunogenicity, such as those carrying HLA-DQA1*05, the use of immunomodulators or the early adoption of alternative therapies could help reduce ADA formation and improve long-term treatment outcomes.

## 6. Ethnic and Geographic Limitations in HLA Studies and Molecular Pathways Linking HLA-DQ Genotypes to Immunogenicity and Therapeutic Resistance in IBD

HLA studies provide key insights into genetic predispositions for inflammatory bowel diseases (IBDs), but they are often limited by ethnic and geographic variability. The frequency and types of HLA alleles differ significantly across different populations. For example, certain HLA alleles, such as HLA-DQ2 and HLA-DQ8, are more prevalent in populations of European descent and are strongly associated with celiac disease, while others, like HLA-DRB1*01, are more common in East Asian populations and are linked to diseases like Crohn’s disease. These differences in allele distribution can lead to challenges in applying study results to broader populations.

Furthermore, many large genetic studies have been conducted predominantly in populations of European descent, creating potential bias in understanding the role of HLA in diseases of other ethnic groups. For example, a study that included nearly 10,000 DNA samples from East Asian, Indian, and Iranian populations, alongside an existing set of 86,640 samples from Europe, North America, and Oceania, showed that genome regions influencing IBD risk are similar worldwide, suggesting that the biology underlying the disease is also consistent.

The HLA-DQA1*05 genotype has a specific structure that allows for more efficient presentation of therapeutic antibody fragments (infliximab and adalimumab) to naïve CD4+ T cells. Due to the unique shape of the peptide-binding groove of this allele, there is an increased likelihood that specific drug fragments will be recognized as foreign antigens, leading to the stimulation of T helper cells (Th cells) and further aiding B cells in the production of high-affinity neutralizing antibodies (ADAs) of the IgG class [86,87].

The key molecular mechanism lies in the fact that the HLA-DQA1*05 allele, when combined with a specific DQB1 chain, forms a peptide-binding groove with a specific electrochemical conformation. This specificity enables better presentation of peptides derived from biological drugs to CD4+ T cells. As a result, T cells assist specific B lymphocytes in secondary lymphoid organs, triggering B-cell activation and their differentiation into plasma cells that produce ADAs [88,89].

**Figure 3:**
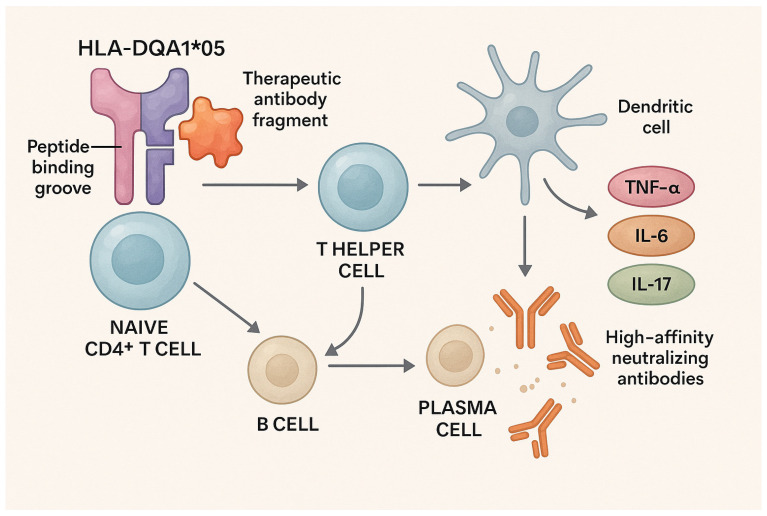
Mechanism of HLA-DQA105-associated immunogenicity and anti-TNF therapy failure. This illustration shows how the HLA-DQA1*05 allele enhances antigen presentation of therapeutic antibodies (infliximab and adalimumab) to naïve CD4+ T cells, promoting T helper and B-cell activation, ADA production, dendritic cell polarization, and cytokine release—contributing to therapy refractoriness in IBD.

Additionally, the interaction between these molecules and dendritic cells influences the immune microenvironment of the intestinal mucosa. In carriers of HLA-DQA1*05, dendritic cells often favor a pro-inflammatory polarization (Th1/Th17 instead of Treg cells), which contributes to a more persistent inflammatory environment and a reduced response to therapy [90,91]. Furthermore, HLA-DQ molecules influence the regulation of inflammatory cytokine expression, such as TNF-α, IL-6, and IL-17, which further contribute to local inflammation and reduced efficacy of biological therapies (Figure 3) [92].

The association with therapy refractoriness lies in the generation of high-titer ADAs that reduce the concentration of free active drugs. This decrease in drug concentration below therapeutic levels leads to secondary LOR. Molecularly, the formation of ADA–drug complexes reduces the bioactivity of the drug, accelerates its elimination from circulation, and hinders the achievement of remission [93,94]. Moreover, ADAs can directly interfere with Fc receptors and reduce drug efficacy through additional immune mechanisms, such as accelerated opsonization and elimination [74].

Studies have shown that the HLA-DQA1*05 genotype carries a two-fold higher risk of secondary LOR during infliximab or adalimumab therapy, which is directly related to its impact on intensified ADA production and reduced drug levels in serum [95]. In contrast, genotypes such as HLA-DQA1*03 have a potential protective effect, reducing the likelihood of ADA formation due to a different molecular groove structure that less efficiently presents biological drug fragments to immune cells [96].

Due to these clear molecular effects, modern approaches to treating IBD patients propose proactive genetic testing for HLA-DQ prior to starting biological therapy, followed by proactive therapeutic monitoring of drug levels and ADAs. Combining this with immunomodulatory drugs (e.g., azathioprine) may further reduce this molecularly mediated risk of immunogenicity and secondary LOR [97,98]. Additionally, new therapeutic strategies, such as tolerogenic vaccines and immune desensitization specific to patients with high-risk HLA-DQ genotypes, are being explored as a promising future direction for personalized approaches to treating inflammatory bowel diseases [99].

Table 3 summarizes the mechanistic links between carrying HLA-DQA1*05 and the development of immunogenicity to anti-TNF agents.

## 7. HLA-Linked SNPs as Biomarkers of Anti-TNF Treatment Resistance

The HLA-DQA1*05 variant (rs2097432) has been investigated as a predictor of response loss to tumor necrosis factor antagonists. The speculation comes from recent clinical trials and GWASs, which have associated the variant with greater affinity for ADA formation [100]. The PANTS study strengthens this hypothesis. The study included 1240 bio-naïve CD patients initiating infliximab or adalimumab; carriers of at least one HLA-DQA1*05 allele were at greater risk of ADA formation, leading to subsequent LOR [39]. Ninety percent of HLA-DQA1*05-positive patients on infliximab monotherapy developed ADAs by the end of the first year of treatment, compared to only 25% of the non-carrier pool, which were using adalimumab combotherapy with an immunomodulator.

The HLA-DRB9 variant, with SNP rs239585, has been associated with a lack of response to infliximab in a pediatric population [101]. Both HLA variants, rs2097432 and rs2395185, were associated with LOR to infliximab, as shown in a multicentric, ambispective trial in a large pediatric IBD cohort [102].

The HLA-DRB1*03 allele was associated with developing an immunogenicity to infliximab in IBD, as shown by Billiet et al. [103]. Key HLA alleles associated with differential response to anti-TNF therapy are summarized in Table 4.

## 8. Gut Microbiota Signatures as Determinants of Refractoriness to Anti-TNF Therapy in IBD

Intestinal microbiota composition as a predictor of treatment response needs to be observed together with clinical and endoscopic or biochemical markers due to its complexity [104].

Few longitudinal studies associated the intestinal microbiota composition with anti-TNFα treatment response. A study by Kolho et al. examined treatment response to anti-TNF α in eleven IBD patients from the pediatric population (six responders and five non-responders) at week 6. The outcome was measured by FCal. Response to anti-TNFs at week 6 was observed in patients who were, at baseline, characterized by a higher relative abundance of five bacteria species, including Bifidobacterium, Clostridium colinum (Clostridium cluster IV), Eubacterium rectale, uncultured Clostridiales, and Vibrio, and a lower relative increase in Streptococcus mitis [105]. A reduction in Faecalobacterium prausnitzi prior to anti-TNF induction was observed in the treatment of non-responders in two separate studies from Emory and Gothenburg University [106,107]. A depleted concentration of F prausnitzii has been associated with early recurrence of CD after discontinuation of anti-TNF-α therapy [108].

Besides changes in the number of beneficial bacteria, one of the factors that also affects response to anti-TNFs in patients with IBD is a higher abundance of potentially harmful bacteria, as observed in some studies. Higher concentrations of Fusobacterium and Veillonella have been verified in anti-TNFα non-responders at baseline [106]. Akkemansia also showed a negative correlation with anti-TNFα response. Shaw et al. exhibited a higher relative abundance at baseline of Akkermansia in non-responders compared to responders. Although this bacterial species is generally known for its positive effects, higher concentrations are not always associated with benefits [109,110]. Apart from the composition of microbiota, response to treatment with anti-TNFs is also dependent on the expression patterns of antimicrobial peptides. Differences in mucosal antimicrobial peptide expressions were observed by Magnusson et al. in UC responders and non-responders to anti-TNFα treatment [107]. As mentioned at the beginning of this chapter, there are many cellular, molecular, genetic, and epigenetic factors that influence therapeutic response. During the decision-making process about which therapy to use, all of these factors/predictors should be taken into account in order to select an effective treatment and maximize the effects of therapy. Finally, as the philosophy of IBD shifts toward precision and personalized medicine, more predictors with greater accuracy are needed to ensure selecting the best therapy for a given patient.

Table 5 provides an overview of microbial taxa associated with favorable or unfavorable anti-TNF response.

## 9. Integrating HLA Genotyping into Pre-Therapeutic Assessment for Anti-TNF Agents

Recent research on HLA testing indicates its increasing application in personalized medicine, especially in the fields of immunology and transplantation [96]. The HLA system plays a key role in regulating the immune response, and certain variants can influence the predisposition to autoimmune diseases and the response to therapy [96,111]. The allele most commonly used for testing before initiating anti-TNF therapy is HLA-DQA105*. Previous studies have shown that carrying this allele increases the risk of developing ADAs, which can reduce the effectiveness of treatments such as infliximab and adalimumab [96,111]. Pretreatment genetic testing for HLA-DQA105* may help personalize therapy selection and optimize the use of immunomodulators to minimize risks and maximize response [112,113].

Future research directions in HLA testing should focus on its growing role in personalized medicine, transplantation, and immunotherapy, with several key areas of research. One major focus is the identification of HLA variants that can predict individual responses to therapeutic interventions, particularly in autoimmune disorders and oncology, enabling the development of more targeted and effective treatment strategies [39]. In addition, HLA typing remains an integral part of optimizing organ transplantation, as researchers continue to refine methods for matching donors and recipients to reduce the likelihood of rejection and improve long-term graft survival [114]. In the field of immunotherapy, HLA testing is increasingly used to identify patients who may benefit significantly from innovative treatments, such as CAR-T therapy and cancer vaccines, thereby facilitating more precise and personalized therapeutic approaches [114,115]. Furthermore, ongoing studies are investigating the complex relationships between specific HLA alleles and the pathogenesis of autoimmune diseases, including rheumatoid arthritis, multiple sclerosis, and type 1 diabetes, to elucidate genetic predispositions and inform preventive and therapeutic strategies [102,116]. Together, these research efforts underscore the potential of HLA testing as a cornerstone of personalized medicine, driving advances that enhance treatment efficacy, minimize adverse outcomes, and improve patient-specific health outcomes across various medical disciplines [102,113].

When HLA testing reveals an unfavorable profile for anti-TNF therapy, indicating an increased risk of developing ADAs and subsequent loss of therapeutic efficacy, alternative treatment strategies that offer comparable disease control with a lower likelihood of immunogenicity should be carefully considered. One widely used approach is to switch to other classes of biologic agents that modulate different immune pathways, such as IL-6 receptor inhibitors such as tocilizumab, which are often used in rheumatoid arthritis, or IL-12/23 and IL-17 inhibitors, which play a significant role in the treatment of psoriasis, psoriatic arthritis, and Crohn’s disease in cases where anti-TNF therapy has proven ineffective [96,116,117,118]. In addition, T-cell modulators such as abatacept, which interfere with essential mechanisms of T-cell activation, represent another viable alternative in the treatment of autoimmune diseases [117,118]. In addition to biologic therapies, small-molecule inhibitors such as JAK inhibitors, including tofacitinib and baricitinib, have attracted increasing attention due to their ability to modulate intracellular signaling pathways rather than directly targeting cytokines, thus offering therapeutic potential in settings where anti-TNF therapy is associated with excessive immunogenic risk [118,119]. Another strategy involves combination therapies that integrate anti-TNF agents with immunosuppressants such as methotrexate, a method designed to attenuate the formation of ADAs and potentially maintain long-term treatment efficacy, although this requires careful clinical monitoring to manage the associated risks [120]. The optimal therapeutic regimen must ultimately be individualized based on a comprehensive assessment of the patient’s overall clinical profile, which includes the type of disease, previous therapeutic responses, comorbidities, and genetic predispositions [121,122]. In this context, multidisciplinary collaboration involving specialists from rheumatology, gastroenterology, dermatology, and immunology ensures that the choice of treatment is not only biologically informed but also holistically tailored to achieve the most favorable clinical outcomes for each patient [96,120,123].

The integration of advanced therapeutic selection methods enhances alignment with patients’ immunogenetic profiles while reducing costs and adverse effects linked to treatment failure. Radiogenetic assays, particularly HLA typing, are increasingly pivotal in developing personalized regimens, allowing for more precise therapy adjustments [124]. Ongoing research aims to refine the predictive role of specific HLA alleles in treatment response, facilitating optimized preclinical decisions, whether through biologic alternatives, small-molecule inhibitors, or combination therapies, to improve efficacy and patient outcomes [122,124].

HLA testing before initiating anti-TNF therapy opens the door to a personalized approach to the treatment of inflammatory diseases such as rheumatoid arthritis, Crohn’s disease, and psoriasis [124]. Although anti-TNF drugs are very effective in controlling inflammation, one of the challenges to the long-term efficacy of these therapies is the development of anti-drug antibodies, which can lead to LOR and reduced safety [39]. Studies have shown that certain HLA variants (e.g., HLA-DQA1*05 in certain populations) may be associated with an increased risk of immunogenicity in patients receiving these drugs [39,118]. The results of these studies suggest that the identification of such genetic markers could help physicians recognize patients who are more likely to develop anti-drug antibodies and take additional steps, such as the introduction of complementary immunosuppressive therapies, to optimize long-term response to treatment. However, although these findings are promising, routine HLA testing before initiating anti-TNF therapy has not yet been widely implemented in clinical practice [118,121]. The evidence, although growing, requires further validation through large, controlled studies to standardize testing protocols and define precise clinical guidelines. Until then, the application of HLA testing remains largely limited to specific situations or as part of research projects, where the aim is to individualize therapy in order to maximize benefits and minimize the risk of side effects [102,123].

From a personalized medicine perspective, the potential use of HLA testing represents a significant step toward improving clinical practice. By combining genetic information with clinical data, it is possible to create individualized treatment plans [39,118]. This may not only improve response to therapy but also reduce the costs associated with eventual restarting or adjusting therapy due to loss of efficacy. As evidence accumulates and testing techniques improve, it is possible that HLA testing will become an integral part of the protocol before initiating anti-TNF therapy [123].

This topic brings us to a broader reflection on the future of immunotherapy and personalized medicine. In addition to anti-TNF therapy, similar approaches are already being used in oncology and transplant medicine, where a patient’s genetic profile plays a key role in choosing the optimal therapeutic modality and minimizing adverse reactions [96,120,122].

## 10. Future Perspectives

Understanding the genetic factors influencing the response to anti-TNF therapy is crucial for personalizing treatment in patients with IBD. While previous studies have highlighted the significance of HLA-DQA1*05 alleles in predicting immunogenicity and secondary LOR to anti-TNF agents, further research is necessary to validate these findings and elucidate the underlying mechanisms. Prospective studies are needed to confirm the association between carrying HLA-DQA1*05 and the development of ADAs and secondary LOR in IBD patients. Integrating HLA-DQA1*05 genotyping into clinical practice could enable clinicians to identify patients at increased risk of immunogenicity and secondary LOR, allowing for tailored therapeutic strategies. For example, combining anti-TNF therapy with immunomodulators such as azathioprine or mercaptopurine may reduce the risk of ADA formation in HLA-DQA1*05 carriers. Developing personalized treatment plans based on HLA genotyping could involve adjusting drug dosages, selecting alternative biologic agents, or incorporating adjunctive therapies to enhance treatment efficacy and minimize adverse effects. For instance, patients with HLA-DQA1*05 alleles who cannot tolerate immunomodulators might benefit from monotherapy with adalimumab, which has a lower immunogenic potential compared to infliximab. Long-term studies assessing the impact of HLA-DQA1*05 genotyping on clinical outcomes, including remission rates, hospitalization frequency, and quality of life, are essential to determine the utility of genetic testing in guiding anti-TNF therapy decisions. Current research focuses on identifying new therapeutic targets in inflammatory bowel diseases (IBDs), with particular emphasis on molecules associated with the HLA system. One significant study through Mendelian randomization identified six plasma proteins (FCGR2A, IL18R1, MST1, HGFAC, IL12B, and ANGPTL3) as potential therapeutic targets for IBD and its subtypes [125]. Furthermore, new clinical trials are investigating the efficacy of molecules affecting HLA-related pathways. For example, the investigation of TL1A inhibitors, such as duvakitug, has shown promising results in achieving clinical remission in patients with ulcerative colitis [126]. Such research could provide evidence to support the incorporation of HLA genotyping into routine clinical practice, ultimately leading to more effective and individualized treatment approaches for IBD patients.

## 11. Conclusions

HLA genotyping represents a promising frontier in the personalization of IBD therapy. Beyond their established roles in disease susceptibility, specific HLA alleles, particularly HLA-DQA1*05, are now recognized as strong predictors of immunogenicity to anti-TNF agents. The ability to identify individuals at increased risk of treatment failure before therapy initiation provides a compelling rationale for preemptive HLA screening in clinical practice. Incorporating HLA data into pharmacogenetic panels may guide therapeutic choices, inform the need for concomitant immunosuppression, and ultimately improve treatment durability. Future prospective studies and cost-effectiveness analyses will be critical to validate and operationalize these approaches, paving the way toward truly individualized IBD management.

## Figures and Tables

**Table 1:** HLA associations in IBD.

HLA Allele/Haplotype	Associated Condition	Reported Effect
HLA-DRB1*01:03	UC	Increased severity, risk of hospitalization, and colectomy
HLA-DRB1*13 (1309, 1320, 1325, 1329)	UC	Associated with pancolitis, surgery, and extraintestinal manifestations
HLA-DRB1*08	UC	Associated with extensive disease
HLA-DRB1*09	UC	Later age of diagnosis
HLA-DRB1*1502	UC	Consistently associated with disease across populations
HLA-DRB1*13:01	UC	Associated with extensive colitis in pediatric-onset UC
rs17188113	UC	Genetic risk marker; female predominance; pediatric onset
HLA-B44	UC	Linked to type II arthritis and erythema nodosum
TNF-1031C	UC	Associated with type II arthritis
HLA-DRB1*0450	CD	Positive association with disease
HLA-DRB1*1502	CD	Protective effect
HLA-DRB1*0405-DQB1*0401	CD	Associated with fistulizing phenotype; ileocecal involvement
HLA-G, MICA/MICB	CD	Associated with disease phenotype
HLA-DRB1*0103	CD	Linked to arthritis and uveitis
HLA-B*44	CD	Associated with extraintestinal manifestations
HLA-B27	CD	Linked to spondyloarthropathy

**HLA**—human leukocyte antigen; **UC**—ulcerative colitis; **CD**—Crohn’s disease; **TNF**—tumor necrosis factor; **MICA/MICB**—MHC class I chain-related gene A/B; **MHC**—major histocompatibility complex.

**Table 2:** Genetic variants affecting anti-TNF pharmacokinetics.

Gene/SNP	Effect on Pharmacokinetics
*ATG16L1* (rs7587051, rs143063741)	Lower infliximab trough levels
*C1orf106* (rs442905, rs59457695)	Variable levels (lower and higher)
*IL6* (rs10499563)	Higher infliximab trough levels
*TLR2* (rs1816702)	Higher adalimumab levels
*NOD2* (rs2066844/45/47)	Dose intensification needed

**SNP**—single-nucleotide polymorphism; **TNF**—tumor necrosis factor; ***ATG16L1***—autophagy related 16 like 1; ***C1orf106***—chromosome 1 open reading frame 106; ***IL6***—interleukin 6; ***TLR2***—toll-like receptor 2; ***NOD2***—nucleotide-binding oligomerization domain-containing protein 2; **trough level**—lowest serum concentration of a drug before the next dose.

**Table 3:** Mechanistic role of HLA-DQA1*05 in anti-TNF therapy immunogenicity.

Mechanism	Description	Consequence
Preferential peptide presentation	HLA-DQA1*05 has a unique binding groove that efficiently presents infliximab/adalimumab fragments to CD4+ T cells.	T-cell activation and B-cell help
B-cell stimulation	Activated Th cells promote differentiation of B cells into plasma cells producing ADAs.	High-titer ADA production
Pro-inflammatory dendritic cell polarization	HLA-DQA1*05 carriers show DC skewing toward Th1/Th17 profiles instead of Treg.	Chronic inflammation and therapy refractoriness
Reduced Treg-mediated suppression	Imbalance between effector and regulatory T cells.	Enhanced ADA generation and sustained inflammation
Upregulation of pro-inflammatory cytokines	Increased TNF-α, IL-6, and IL-17 expression.	Reduced drug levels and effectiveness
Formation of ADA–drug immune complexes	Immune complexes neutralize drug activity and accelerate clearance.	Loss of therapeutic response (secondary LOR)

**ADAs**—anti-drug antibodies; **DCs**—dendritic cells; **Th**—T helper cells; **Treg**—regulatory T cells; **TNF-α**—tumor necrosis factor alpha; **IL**—interleukin; **LOR**—loss of response.

**Table 4:** HLA alleles associated with IBD and anti-TNF therapy response.

HLA Allele	Associated Disease	Impact
HLA-DRB1*01:03	UC	Increased severity, risk of surgery
HLA-DRB1*1502	UC	Consistent association across populations
HLA-DQA1*05	CD and UC	Increased ADA formation, therapy failure
HLA-DRB1*03	IBD	Immunogenicity to infliximab
HLA-DRB9 (rs239585)	IBD (pediatric)	Loss of response to infliximab

**HLA**—human leukocyte antigen; **IBD**—inflammatory bowel disease; **UC**—ulcerative colitis; **CD**—Crohn’s disease; **ADAs**—anti-drug antibodies; **TNF**—tumor necrosis factor.

**Table 5:** Gut microbiota predictors of anti-TNF therapy response.

Microbial Feature	Association with Response
*Faecalibacterium prausnitzii ↓*	Non-response, recurrence
*Bifidobacterium ↑*	Response (pediatric)
*Fusobacterium ↑*	Non-response
*Akkermansia ↑*	Negative predictor
*Streptococcus mitis ↑*	Non-response (↑ at baseline)

**↑**—increased abundance; **↓**—decreased abundance; **TNF**—tumor necrosis factor.

## Data Availability

Data are contained within the article.

## Abbreviation

ADAs	Anti-drug antibodies
ADA (drug)	Adalimumab
APC	Antigen-presenting cell
ATG16L1	Autophagy-related 16-like 1
BSA	Body surface area
CAR-T	Chimeric antigen receptor T-cell therapy
CD	Crohn’s disease
C1orf106	Chromosome 1 open reading frame 106
DC	Dendritic cell
DQ	HLA-DQ locus
DR	HLA-DR locus
Fc	Fragment crystallizable region
FcγR	Fc gamma receptor
FCGRT	Neonatal Fc receptor transporter gene
FCal	Fecal calprotectin
GWASs	Genome-wide association studies
HLA	Human leukocyte antigen
IFX	Infliximab
IL	Interleukin
IL-6	Interleukin 6
IL-17	Interleukin 17
IL-23	Interleukin 23
JAK	Janus kinase
LOR	Loss of response
MHC	Major histocompatibility complex
MICA/MICB	MHC class I chain-related gene A/B
mAbs	Monoclonal antibodies
NOD2	Nucleotide-binding oligomerization domain-containing protein 2
PBMC	Peripheral blood mononuclear cell
pTDM	Proactive therapeutic drug monitoring
SERENE	Study of the Efficacy and Safety of Adalimumab
SNP	Single-nucleotide polymorphism
TCR	T-cell receptor
Th cell	T helper cell
TLR2	Toll-like receptor 2
TNF	Tumor necrosis factor
TNF-α	Tumor necrosis factor alpha
Treg	Regulatory T cell
UC	Ulcerative colitis
VNTR	Variable number tandem repeat
